# A Facile Method for the Fabrication of the Microneedle Electrode and Its Application in the Enzymatic Determination of Glutamate

**DOI:** 10.3390/bios13080828

**Published:** 2023-08-18

**Authors:** Mahmoud Amouzadeh Tabrizi

**Affiliations:** Electronic Technology Department, Universidad Carlos III de Madrid, 28911 Leganés, Spain; mamouzad@ing.uc3m.es or mahmoud.tabrizi@gmail.com

**Keywords:** microneedles, electrochemical biosensor, glutamate oxidase, neurotransmitters, glutamate

## Abstract

Herein, a simple method has been used in the fabrication of a microneedle electrode (MNE). To do this, firstly, a commercial self-dissolving microneedle patch has been used to make a hard-polydimethylsiloxane-based micro-pore mold (MPM). Then, the pores of the MPM were filled with the conductive platinum (Pt) paste and cured in an oven. Afterward, the MNE made of platinum (Pt-MNE) was characterized using cyclic voltammetry (CV), electrochemical impedance spectroscopy (EIS), and scanning electron microscopy (SEM). To prove the electrochemical applicability of the Pt-MNE, the glutamate oxidase enzyme was immobilized on the surface of the electrode, to detect glutamate, using the cyclic voltammetry (CV) and chronoamperometry (CA) methods. The obtained results demonstrated that the fabricated biosensor could detect a glutamate concentration in the range of 10–150 µM. The limits of detection (LODs) (three standard deviations of the blank/slope) were also calculated to be 0.25 µM and 0.41 µM, using CV and CA, respectively. Furthermore, the Michaelis–Menten constant (K_M_^app^) of the biosensor was calculated to be 296.48 µM using a CA method. The proposed biosensor was finally applied, to detect the glutamate concentration in human serum samples. The presented method for the fabrication of the mold signifies a step further toward the fabrication of a microneedle electrode.

## 1. Introduction

Nowadays, thanks to the advances in nano/micro-technology, the fabrication of wearable medical devices is no longer an unattainable dream. It opens a new chapter in the field of bioengineering. Wearable medical devices are smart tools that can be used for drug/vaccine delivery [1,2], biomolecular sensing [3,4], etc. Bimolecular sensing, tattoo sensors [5,6], and microneedle-based sensors [7,8] are the most well-known wearable sensors,and are designed to measure the molecules attached to the skin, to detect molecules. The tattoo sensors are applied for the determination of the molecules in sweat, such as small molecules [4,9,10] and ions [11,12]. On the contrary, microneedle electrodes (MNEs) are designed to measure the molecules in the interstitial fluid (ISF).

MNEs have been used widely in the real-time monitoring of biomolecules, such as glucose [13,14,15], lactate [16], alcohol [17], urea [18], drugs [19], etc. [20,21,22].

Up until now, several techniques have been reported in the literature for the fabrication of the MNE, such as photolithography [23], laser cutting [24], laser ablating [25], electroplating [26], droplet-born air blowing [27], atomized spraying [28], pulling pipettes [29], and micro-molding [30]. Among them, the micro-molding technique has been used extensively. The micro-mold can be made using a laser drill of polydimethylsiloxane (PDMS) [31], a 3D printer [32], and the spark erosion of aluminum [33]. All of these techniques are expensive anddangerous, and require operators with a high level of experience. Thus, there is a need to explore new micro-mold fabrication methods to be used by all researchers. In this research work, a self-dissolving microneedles patch (SD-MNP) was used to fabricate a mold. The SD-MNPs are cheap and commercially available in medical stores. The SD-MNP was used as a template to fabricate a micro-mold using hard-polydimethylsiloxane (h-PDMS),and then it was applied to the fabrication of the MNE using platinum paste. To evaluate the possible analytical applications of the described method, the platinum-based MNE (Pt-MNE) was applied to the electrochemical sensing of glutamate.

Glutamate is a neurotransmitter molecule [34], and an abnormal glutamate level is associated with several neurologicaldiseases, such as Parkinson’s [35] and Alzheimer’s [36]. Hence, the fabrication of a biosensor to detect glutamate in human real samples is important to diagnosingthese diseases in the early stages. Up until now, several biosensors have been designed for glutamate detection, such as aptamer-based [37,38] and enzymatic-based [39,40,41,42] sensors. Among them, enzymatic-based sensors are more interesting, due to their low cost, width response range, and stability.

Joseph Wang and his team reported the first MNE for glutamate biosensing in 2011 [43]. Although the analytical performances of the biosensor were good, its limit of detection (LOD) was 10 µM, so it is not suitable for the detection of glutamate in the brain ISF, because the glutamate level in the brain ISF is below 2 µM [44] and it increases to 40–60 μM in human blood [45]. Hence, their sensor can only be used in the detection of glutamate in blood samples.

In this research work, I have fabricated a highly sensitive MNE to detect glutamate in the range of 10–150 µM, with LODs of 0.25 µM and 0.41 µM, using cyclic voltammetry (CV) and chronoamperometry (CA), respectively, making it a good candidate for real-time studies on the brain ISF. In addition, the proposed MNE had a wide linear-response range, high selectivity, and stability.

## 2. Materials and Methods

### 2.1. Reagents and Chemicals

SD-MNPs were purchased from Trouble Dr. Company (Seoul, Republic of Korea). Conductive platinum paste (Pt particle size <15 um) was purchased from Col-Int Tech Company (Irmo, SC, USA). Hard-polydimethylsiloxane (h-PDMS) was purchased from CymitQuimica (Barcelona, Spain). Glutamate oxidase (GLOx, 5 U), glutamate, glucose, lactate, ascorbic acid, uric acid, urea, hydrogen peroxide (H_2_O_2_), hexaammineruthenium(III) (Ru(NH_3_)_6_^3+^), and Nafion 117 were obtained from Sigma–Aldrich (St. Louis, MO, USA). The glutamate assay kit was purchased from Abcam (Waltham, Boston, MA, USA). Phosphate-buffered saline (PBS) was purchased from Thermo Fisher Scientific (Waltham, MA, USA). Epoxy resin and copper tape were purchased from Resin Pro (Barcelona, Spain) and FEPITO (Shenzhen, China), respectively.

### 2.2. Apparatus

The CV and CA studies were performed using a µStat 300 Bipotentiostat (Metrohm-DropSens, Oviedo, Spain), with a conventional three-electrode setup, in which a Pt-MNE/GLOx/Nafion, a platinum wire, and a Ag/AgCl/KCl_saturated_ served as the working, auxiliary, and reference electrodes, respectively. The working potential was utilized in a standard manner, and the output signal was obtained using DropView 8400 software. To record the CA signals, +0.4 V was applied to the working electrode.

High-resolution scanning electron microscopy (HR-SEM) used a double-beam FIB-FEGSEM (Field Emission Gun) Helios NanoLab 600i (FEI Company, Hillsboro, OR, USA). The electrochemical impedance spectroscopy (EIS) studies were performed using an ISX-3 impedance analyzer (Sciospec, Bennewitz, Germany). Typical EIS experiments were presented, in the form of the Nyquist plot,and recorded in a Ru(NH_3_)_6_^3+^ (5 mM, Ph = 7.4) solution as the redox probe. An alternating current (AC) voltage of 10 mV, and a direct current (DC) voltage of −0.15 V were applied over a frequency range of 100 kHz to 0.1 Hz. The EIS data were analyzed using EIS spectrum analyzer (EISSA) software.

### 2.3. Fabrication of the Micro-Pore Mold (MPM)

An SD-MNP was, firstly, attached to the surface of a glass Petri dish with glue, and allowed to dry for 15 min. Then, the h-PDMS mixture was poured on the MNP surface, to cover the Petri dish. The Petri dish was planted inside a vacuum chamber for 1 h (under a negativepressure of 100 mm Hg), to remove any trapped bubbles in the h-PDMS. Subsequently, the Petri dish was transferred to the oven to cure overnight at 80 °C. Finally, the mold was removed from the Petri dish, and immersed inside deionized water, and the water was shaken gently, to remove any broken needles that might be inside the pores. The mold was stored in a clean box when not in use.

### 2.4. Fabrication of the Pt-MNE

The MNM was first attached inside a centrifuge tube (15 mL) with double-sided adhesive tape. The tube was then filled with 5 mL of Pt paste, and centrifuged (5000 rpm) for 5 min, to transfer the Pt paste into the pores of the h-PDMS mold. After that, the sample was transferred to an oven to cure at 120 °C for 2 h. Finally, the dried Pt layer was gently peeled off from the h-PDMS mold. The Pt-MNE was carefully attached to the surface of a clear glass plate. To do this, copper tape was used as a connector between the electrochemical device and Pt-MNE. The copper tape then was covered with an epoxy resin, to isolate it. The Pt-MNE was stored in a clean box when not in use.

### 2.5. Fabrication of the Biosensor

A total of 60.0 µL of GLOx (5.0 U·µL^−1^) was cast on the surface of the Pt-MNE, and allowed to dry at room temperature. To retain the enzyme strongly attached to the surface of the Pt-MNE, GLOx enzymes were linked to each other, via the drop casting of 60.0 µL of glutaraldehyde (3%, 0.1 M PBS) to the surface of the Pt-MNE, andwere left to dry at room temperature. Then, the Pt-MNE was washed with 0.1 M PBS, and 50.0 µL Nafion (0.5%) was then cast on the surface of the Pt-MNE, and allowed to dry at room temperature. Nafion is a biocompatible polymer [46,47], which is widely used in the fabrication of enzyme-based biosensors [48,49].

**Figure 1:**
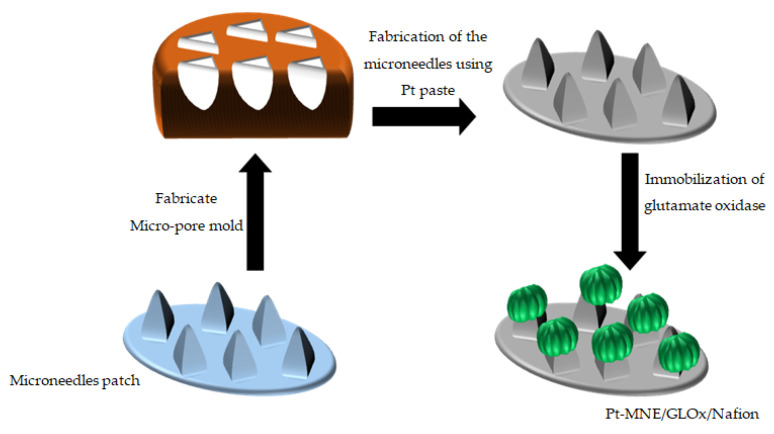
Schematic fabrication of the micropores mold using the microneedle patch, and its application in the fabrication of the Pt-MNE/GLOx/Nafion.

In addition, Nafion not only helps to attachGLOxto the Pt-MNE surface, but also repeals negatively charged molecules, such as ascorbic acid and uric acid, from the Pt-MNE surface that might affect the signal of the biosensor (Pt-MNE/GLOx/Nafion). The Pt-MNE/GLOx/Nafion was finally washed, andwas stored in a refrigerate (4 °C) when not in use. The Pt-MNE/GLOx/Nafion, platinum electrode, and Ag|AgCl were the working electrod, current electrode, and reference electrode, respectively. Figure 1 demonstrates the scematic fabrication process of the microneedle and the biosensor.

## 3. Results and Discussion

### 3.1. Surface Characterization of the Samples

**Figure 2:**
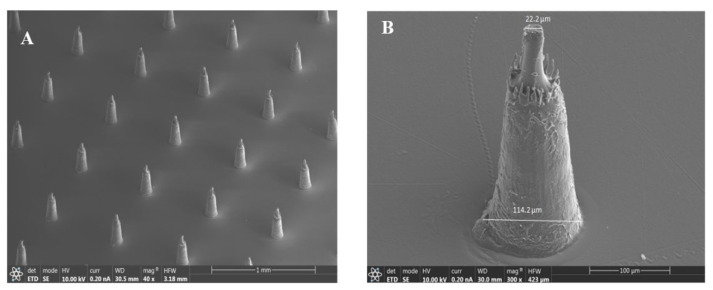

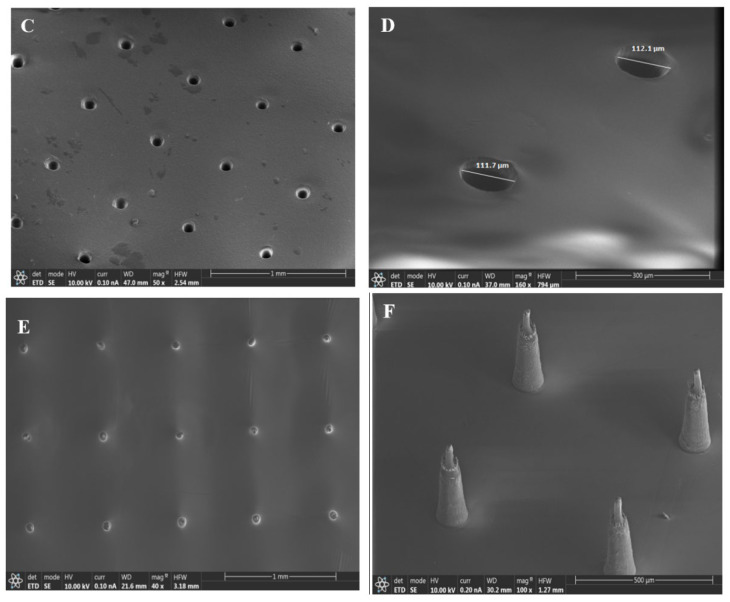
SEM images of the microneedle patch (**A**,**B**), h-PDMS-based mold (**C**,**D**), and PT-MNE (**E**,**F**).

Figure 2 shows the SEM images of the microneedle patch (A, B), the micropores of the h-PDMS (C, D), and the MNE (E, F). As shown in Figure 2A,B, the average length of the microneedle patch is 230 ± 2 µm. The average diameteron the apex and base sides were 22 ± 0.3 and 114 ± 0.6 µm, respectively. Hence, this microneedle patch is a good and cheap sample for making a mold with micropores. Further investigations indicated that the produced mold with h-PDMS had pores with an average diameter of 111 ± 0.4 µm, which is close to the diameter of the base side of the needle (Figure 2C,D), indicating that the mold was fabricated successfully. The mold was then used in the fabrication of the Pt-MNE (Figure 2E,F). As shown in the figures, the Pt-MNE was made of several micro needles, whose average length and diameters from the apex and base sides were 224 ± 3, 19 ± 0.25, and 111 ± 0.4 µm, respectively. As can be seen, the size of the Pt-MNE was shrunk during the curing process of the Pt conductive paste, because Pt conductive paste is made of epoxy resin and it shrinks (in a range of 1–5%) during curing. Contrary to the previous MNEs [18,50,51], all of the proposed microneedles can penetrate the skin, because of their small height and base diameter.

### 3.2. Electrochemical Behavior of Pt-MNE

**Figure 3:**
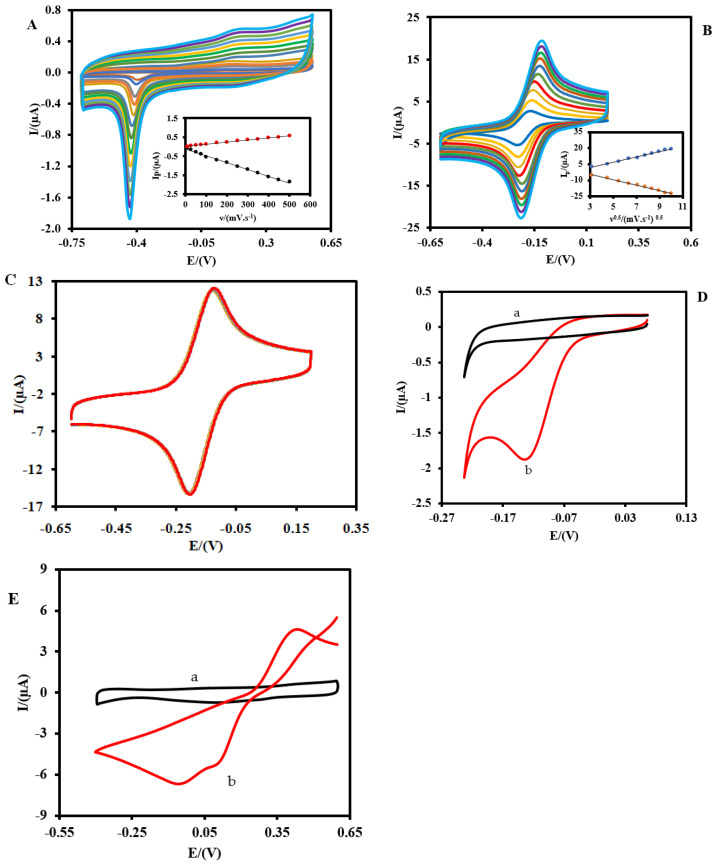
(**A**) CVs of the Pt-MNE in 0.1 M NaOH at various scan rates from 10 to 700 mV·s^−1^ (10, 25, 50, 75, 100, 150, 200, 250, 300, 350, 400, 450, and 500 mV·s^−1^ from inner to outer). Inset: plot of the peak current (I_p_) versus scan rate (ν). (**B**) CVs of the Pt-MNE in 0.1 M PBS containing Ru(NH_3_)_6_^3+^ (5 mM, pH = 7.4) at various scan rates (10, 20, 30, 40, 50, 60, 70, 80, 90, and 100 mV·s^−1^ from inner to outer) Inset: plot of the peak currents versus the square root of the scan rate (ν^1/2^). (**C**) Electrochemical stability of Pt-MNE in 0.1 M PBS containing Ru(NH_3_)_6_^3+^(5 mM, pH = 7.4), at a scan rate of 50 mV·s^−1^. (**D**) CVs of the Pt-MNE in an N_2_-saturated (a) and an O_2_-saturated (b) PBS (0.1 M, pH = 7.4), at a scan rate of 50 mV·s^−1^. (**E**) CVs of the Pt-MNE in the absence (a) and presence (b) of 0.5 mM H_2_O_2_ (0.1 M PBS, pH = 7.4), at a scan rate of 50 mV·s^−1^.

Figure 3A indicates the CVs of the Pt-MNE in 0.1 M NaOH at different scan rates. As shown, a well-defined oxidation peak related to the formation of $PtOx(Pt+H_{2}O\to PtO+2H^{+}+2e^{-}$ Equation (1), and a reduction peak related to the reduction of$\;PtOx(PtOx+2H^{+}+2e^{-}\to Pt+H_{2}O,$ Equation (2) were observed, indicating that the MNE was made of Pt [52]. The oxidation peak and reduction peak currents were linearly proportional to the scan rate (ν), in the range of 10–500 mV·s^−1^ (Figure 3A, inset), due to the surface-controlled redox process. Moreover, the CVs of the Ru(NH_3_)_6_^3+^(5 mM, 0.1 M PBS) as a redox probe were recorded with a Pt-MNE at different scan rates (10–100 mV.s^−1^) (Figure 3B). Unlike the oxidation/reduction of the Pt, the oxidation and reduction peak currents were linearly proportional to the square root of the scan rate (ν^1/2^),due to the diffusion-controlled redox process of the redox probe (Figure 3B, inset). The stability of the signal of Pt-MNE to Ru(NH_3_)_6_^3+^(5 mM, 0.1 M PBS, pH = 7.4) was also investigated (Figure 3C). As shown, the signals did not change notably after 20 potential cycles,indicating the high stability of the Pt-MNE.

The electrochemical responses of the Pt-MNE to the dissolved oxygen (O_2_) and hydrogen peroxide (H_2_O_2_) were studied (Figure 3D,E). Because an oxidase enzyme such as GLOx consumes O_2_ and generates H_2_O_2_ (Equation (3)) [53], both molecules can be detected using electrochemical Pt-based electrodes. GLOx     L-Glutamate + O_2_ + H_2_O → 2-Oxoglutarate + NH_3_ + H_2_O_2_ (Equation (3)).

As shown in Figure 3D, the CVs of the Pt-MNE in a nitrogen-saturated (a) and an O_2_-saturated (b) PBS (0.1 M, pH = 7.4) displayed high electrocatalytic activity in the Pt-MNE toward the O_2_ reduction.

In addition, the results revealed that H_2_O_2_ could be electrochemically oxidized and reduced on the Pt-MNE surface (Figure 3E). As can be seen, the Pt-MNE oxidized 0.5 mM H_2_O_2_ (0.1 M PBS, pH = 7.4) at 0.43 V, and reduced it at two steps, at 0.06 V and 0.07 V. Asthe reduction peaks of H_2_O_2_ and O_2_ were close to each other, and it is difficult to trust the results (because on the one hand, the O_2_ concentration decreases during the enzymatic oxidation of glutamate by GLOx, but on the other hand, the H_2_O_2_ concentration increases), the electrochemical oxidation of the generated H_2_O_2_ was studied during glutamate measurement with the Pt-MNE/GLOx/Nafion.

**Figure 4:**
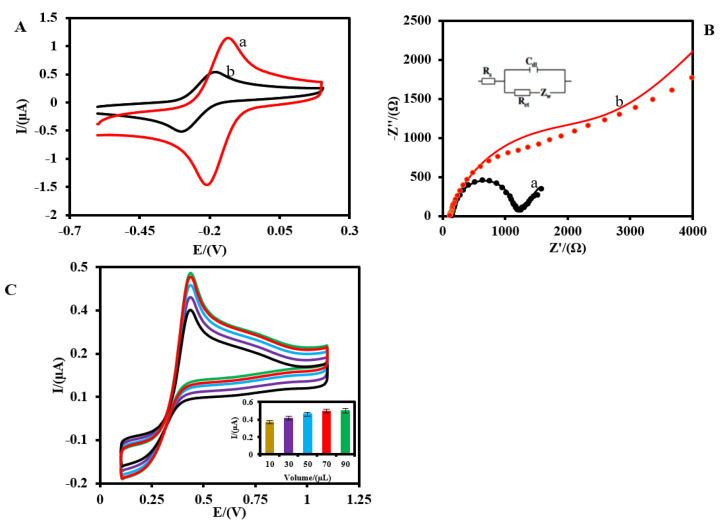
(**A**) CVs of the Pt-MNE (a) and Pt-MNE/GLOx/Nafion (b) in 0.1 M PBS containing Ru(NH_3_)_6_^3+^(5.0 mM, 0.1 M PBS). (**B**) Nyquist plot of the Pt-MNE (a) and Pt-MNE/GLOx/Nafion (b) 0.1 M PBS containing Ru(NH_3_)_6_^3+^(5 mM, pH = 7.4). The set is the equivalent of the equivalent electric circuit compatible with the Nyquist diagrams. R_s_: solution resistance, R_et_: electron transfer resistance, C_dl_: double-layer capacitance, Z_w_: Warburg impedance. The AC amplitude voltage was 10 mV, the DC voltage was −0.17 V, and the frequency range was from 100,000 to 0.1 Hz. The marked lines and solid lines were related to the experimentally obtained spectrum and fitted spectrum, respectively. (**C**) The effect of the different volumes of GLOx enzyme used in the fabrication of the Pt-MNE/GLOx/Nafion to 50.0 µM glutamate in an O_2_-saturated PBS (0.1 M, pH = 7.4). The scan rate was 50 mV·s^−1^.

The effect of the immobilized GLOx enzyme on the electrochemical behavior of Pt-MNE is shown in Figure 4. The CV results indicated that, after the immobilization of the GLOx enzyme (red curve), the intensity of the anodic and cathodic peak currents decreased from 111.0 and −146.0 µA to 55.0 and −51.0 µA, respectively, and the peak-to-peak separation of the Ru(NH_3_)_6_^3+^(5.0 mM, 0.1 M PBS) increased from 58.0 to 102.0 mV (Figure 4A). It indicated that the immobilized GLOx enzyme molecules inhibited the access of Ru(NH_3_)_6_^3+^to the Pt-MNE surface. The EIS method has been also applied to the surface electrochemical characterization of Pt-MNE, before (a) and after (b) the immobilization of the GLOx enzyme (Figure 4B). The typical Nyquist plot includes a semicircle portion at higher frequencies, related to the electron-transfer-limited process, and a linear part at a lower frequency range, representing the diffusion-limited process. The semicircle diameter equals the electron-transfer resistance (R_et_). This resistance controls the electron-transfer kinetics of Ru(NH_3_)_6_^3+^ as the electrochemical probe at the electrode interface. As shown in Figure 4B, the R_et_ value of the Pt-MNE electrode (a) was less in comparison with the Pt-MNE/GLOx/Nafion (b) (1040.1 Ω versus 2315.6 Ω), suggesting that the immobilized GLOx enzyme increased the resistance of the electrode dramatically. The modeling results, with the values of the different parameters and fitting errors, are shown in Figure S1.

The effect of the enzyme on the sensitivity of the biosensor was also investigated using the CV method (Figure 4C) in an O_2_-saturated PBS (0.1 M, pH = 7.4). As can be seen, the sensitivity of the biosensor to 50.0 µM glutamate increased with the increase in the GLOx volume from 10.0 to 70.0 µL, and then it remained unchanged at 90.0 µL of GLOx. Hence, throughout this work, 70 µL of GLOx solution was used in the fabrication of the Pt-MNE/GLOx/Nafion.

### 3.3. The Electrochemical Performance of Pt-MNE/GLOx/Nafion

**Figure 5:**
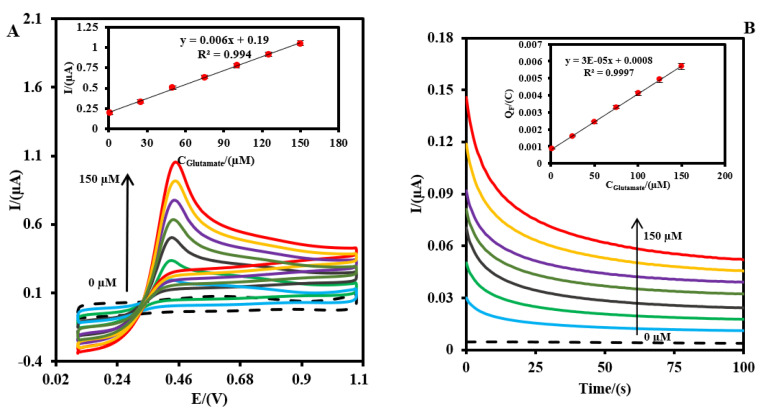

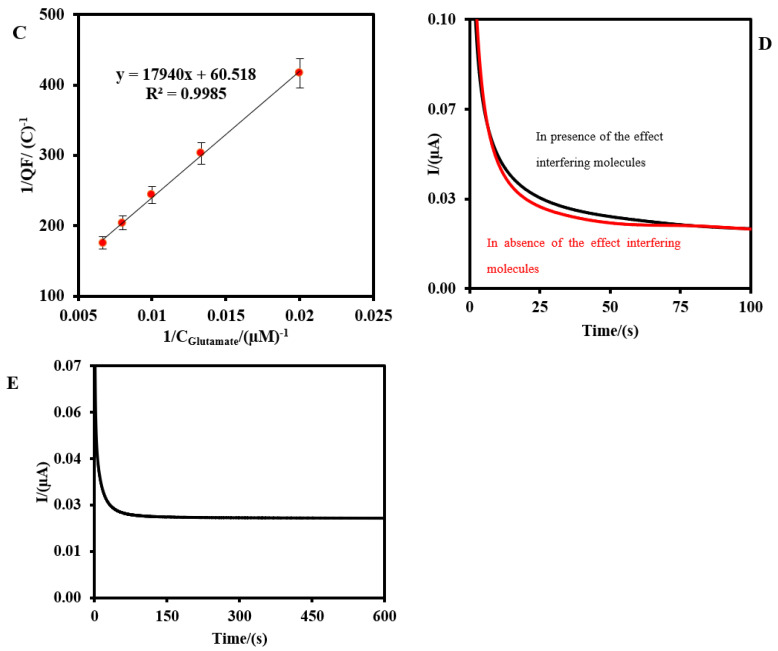
(**A**) CV and (**B**) CA of the Pt-MNE/GLOx/Nafion in an O_2_-saturated PBS (0.1 M, pH = 7.4) containing different amounts of glutamate (0.0, 1.0, 25.0, 50.0, 75.0, 100.0, 125.0, and 150.0 µM, from inner to outer). Inset: plot of the signal versus the concentration of glutamate. (**C**) Lineweaver–Burk plot obtained using the CAM technic. (**D**) Effect of 5mM of the effect-interfering molecules (glucose, lactate, ascorbic acid, uric acid, and urea) (black curve) on the response of the Pt-MNE/GLOx/Nafion to 25.0 µM of glutamate. (**E**) Stability of the signal of the Pt-MNE/GLOx/Nafion to 25.0 µM of glutamate. CVs were recorded at a scan rate of 50 mV·s^−1^. The applied potential to record the CA was +0.4 V.

Figure 5 shows the CVs (A) and CAs (B) of the Pt-MNE/GLOx/Nafion in an O_2_-saturated PBS (0.1 M, pH = 7.4) in the absence (dot line) and the presence (solid lines) of the different concentrations of glutamate. As can be seen in Figure 5A, the oxidation peak current increased as the glutamate level in the sample increased from 1.0 to 150.0 µM. As shown in the inset of Figure 5A, the slope of the calibration plot related to the oxidation of the generated H_2_O_2_ during the reaction of the GLOx enzyme and glutamate increased with a regression equation I_p_ (μA) = +0.006 C_Glutamate_ (µM) +0.19 (Equation (4)), with a correlation coefficient of 0.99 (n = 4).

The glutamate concentration was also measured using the CA method at +0.4 V (versus Ag|AgCl) (Figure 5B). As can be seen, the Faradaic (F) currents related to the oxidation of the generated H_2_O_2_ increased with the increasing glutamate concentration. The Faradaic charge (Q_F_) as a signal versus glutamate increased with a regression equation Q_F_ = +3 × 10^−5^ C_Glutamate_ (C) +8.0 × 10^−4^ (Equation (5)), with a correlation coefficient of 0.99 (n = 4). The Q_F_ values were measured via the integration of the current–time plots in Figure 5B, and using the equation: Q_F_ = Q_total_ − Q_non-Faradic_ (Equation (6)), where Q_non-Faradic_ is the charge value in the absence of glutamate, and Q_total_ is the charge value in the presence of glutamate. The LODs were calculated to be 0.25 and 0.41 µM using CV and CA (three standard deviations of the blank/slope), respectively. The related error bars correspond to the standard deviation for the four measurements of the glutamate. The LOD of the proposed MNE for glutamate was lower than the previous biosensor reported by Wang’s team [43]. It might be because of the good catalytic property of the Pt microparticles (<15 µm) to the H_2_O_2_ that was in the conductive platinum paste.

Via the CA technique, the Michaelis–Menten constant (K_M_^app^) of the Pt-MNE/GLOx/Nafion was calculated to be 296.48 µM from its intercept and the slope of the Lineweaver–Burk equation: $\frac{1}{Q_{m}}=\frac{1}{Q_{max}}+\frac{K_{M}^{app}}{Q_{max}}\times \frac{1}{C_{Glutamate}}$ (Equation (7)) [54], where Q_m_ is the Faradaic charge related to the oxidation of the glutamate, Q_max_ is the maximum charge, and C_glutamate_ is the glutamate concentration. The Lineweaver–Burk plot is shown in Figure 3C. The smaller value of theK_M_^app^ of GLOx indicates that the Pt-MNE provides a higher enzymatic activity to glutamate oxidation and, subsequently, increased the affinity biosensor.

The selectivity of the biosensor to glutamate was also investigated using CA (Figure 5D). As shown, in the presence of 5 mM glucose, lactate, ascorbic acid, uric acid, and urea as the interfering molecules, the response of the Pt-MNE/GLOx/Nafion to 25.0 µM glutamate was changed by 4.3%. The error might be caused by the diffusion of the interfering electroactive molecules to the Pt-MNE surface. The low error value indicated that the Nafion file repealed the negatively charged molecules, such as ascorbic acid, lactic acid, and uric acid from the Pt-MNE/GLOx/Nafion surface during the glutamate measurement.

Furthermore, the stability of the Pt-MNE/GLOx/Nafion to 1.0 µM glutamate was studied for 600 s (Figure 5E). As can be seen, the signal of the biosensor did not change. It proves the capability of the Pt-MNE/GLOx/Nafionin the real-time investigation of the glutamate level in real samples.

To examine the applicability of Pt-MNE/GLOx/Nafionin real sample analysis, we applied it to glutamate determination in human serum samples, using CA. Briefly, 500 µL of normal human serum was mixed with 1.5 mL O_2_ saturated PBS (0.1 M, pH = 7.4), and then analyzed using the Pt-MNE/GLOx/Nafion. After four measurements of the sample, the average glutamate concentration in normal human serum for sample 1 and sample 2 was found to be 45.27 µM and 50.3 µM, respectively. The *t*-test and *p*-test values were reported in Table 1. As can be seen, there are no significant differences between the Pt-MNE/GLOx/Nafion and glutamate assay kit.

The analytical performances of the Pt-MNE/GLOx/Nafionare compared with the other biosensor for glutamate in Table 2. As can be seen, in most terms, the analytical performance of the Pt-MNE/GLOx/Nafion was better than the others.

Finally, the reproducibility of the Pt-MNE/GLOx/Nafion was investigated using four biosensors. The relative standard deviation (R.S.D) was calculated to be 5.1% using CA. All the results indicated that Pt-MNE/GLOx/Nafion is a good candidate to detect glutamate in real samples.

## 4. Conclusions

In conclusion, a simple, low-cost, and rapid method for the fabrication of the electrochemical MNE has been developed, using a commercial microneedle patch for clinical applications. Moreover, an enzymatic electrochemical biosensor has been fabricated to detect glutamate in the range of 10–150 µM, with LODs of 0.25 µM and 0.41 µM, using CV and CA, respectively, and with negligible interference from other molecules. The proposed electrochemical Pt-MNE-based biosensor showed excellent electrocatalytic activity for the H_2_O_2_generated by theGLOx enzyme, based on which the oxidization current of the generated H_2_O_2_ increased with the increasing glutamate in the sample. The Pt-MNE-based biosensor leads to a sensitive, selective, and cost-effective diagnostic medical device. Furthermore, the Pt-MNE-based biosensor showed excellent applicability to detect glutamate in human serum samples. Future research should be conducted, to make it a wearable biosensor.

## Figures and Tables

**Table 1:** Comparison of the obtained results between thePt-MNE/GLOx/Nafion using the CA technique, and a glutamate assay kit, for glutamate.

Sample	Obtained Value Using Pt-MNE/GLOx/Nafion/ (µM)	Mean/(µM)	Standard Deviation	Count	Standard Error of Mean	Degree of Freedom	Hypothesized Mean/(µM)	T-Value ^†^	*p*-Value	Obtained Value Using Glutamate Assay Kit/ (µM)
1	45; 45.5, 45.2; 45.4	45.27	0.22	4.0	0.11	3.0	45.0	2.48	0.089	45
2	50.1; 50.5; 50.2; 50.4	50.3	0.18	4.0	0.09	3.0	50.0	3.28	0.046	50.0

^†^: two-tailed test 0.

**Table 2:** Comparison of the analytical performance of the Pt-MNE/GLOx/Nafion with other biosensors for glutamate.

Biosensor	Method	Linear Range	Limit of Detection	References
Pt/P(o-PD)/GLOx	CA	20–140 µM	10 µM	[43]
Pt wire/P(o-PD)/GLOx/Chit/ASOx	CA	5–35 µM	0.59 µM	[40]
Gold Electrode/PNAI/PPyNps	CA	0.02–400 µM	0.1 nM	[55]
MWCNT-Chit-MB/GLDH-NAD-Chit-MB/MWCNT-Chit -MB	CA	7.5–105 μM	3.0 μM	[56]
Pt wire/GLOx	CA	2.5–450 μM	1.0 μM	[57]
SPC/PB/GLOx/Chit	CA	25–300 μM	9.0μM	[58]
SPCE/AuNP/GluBP	CV	0.1–0.8 mM	0.15 mM	[59]
Gold electrode/Chit/AuNP/cMWCNT/GluOx	CA	5–500 μM	1.6 μM	[60]
GCE/PEI-MWNTs/bacteria-Gldh/Nafion	CA	10 μM–1 mM and 2–10 mM	2.0 μM	[61]
SPCE/Chit-AA-CDs/GLDH	CA	11–125 μM	3.0 μM	[62]
GCE/PtNP@MXene-Ti3C2Tx/Chi	CA	10–110	0.45	[63]
Gold electrode/Chi/GO/Au NPs/GluOx	DPV	0.2–1.4 mM	0.023 mM	[64]
SPPtE/PPYox/GLOx	CA	0.005–1.0 mM	1.8 μM	[65]
Pt-MNE/GLOx/Nafion	CV CA	1–150 μM 1–150 μM	0.25 μM 0.41 μM	This work

P(o-PD): poly o-phenylenediamine; Chit: chitosan; AsOx: ascorbate oxidase; AMP: amperometry; PpyNps: polypyrrolenanoparticles; PNAI: polyaniline; MWCNT: multiwalled carbon nanotubes; GLDH: glutamate dehydrogenase; MB: Meldola’s Blue; NAD: nicotinamide adenine dinucleotide; PB/SPC: screen-printed Prussian blue nanocube microchip; GluBP: glutamate binding protein; AuNP: gold nanoparticle; AA-CDs: carbon nanodots modified with azure A; GCE: glassy carbon electrode; PtNP: Pt nanoparticles MXene-Ti3C2Tx: two-dimensional nanomaterial; DPV: differential pulse voltammetry; GO: graphene oxide; PPYox: overoxidized polypyrrole film; SPPtE: screen-printed platinum electrodes.

## Data Availability

Supplementary Data associated with this article can be found in the online version, including Figure S1.

## Supplementary Materials

The following supporting information can be downloaded at: https://www.mdpi.com/article/10.3390/bios13080828/s1, Figure S1: Images of the modeling results with values of the different parameters.

## References

[1] Kim Y.-C., Park J.-H., Prausnitz M.R. (2012). Microneedles for drug and vaccine delivery. Adv. Drug Deliv. Rev..

[2] Yang J., Liu X., Fu Y., Song Y. (2019). Recent advances of microneedles for biomedical applications: Drug delivery and beyond. Acta Pharm. Sin. B.

[3] Dardano P., Rea I., De Stefano L. (2019). Microneedles-based electrochemical sensors: New tools for advanced biosensing. Curr. Opin. Electrochem..

[4] Wang J., Lu Z., Cai R., Zheng H., Yu J., Zhang Y., Gu Z. (2023). Microneedle-based transdermal detection and sensing devices. Lab Chip.

[5] Bandodkar A.J., Jia W., Yardımcı C., Wang X., Ramirez J., Wang J. (2015). Tattoo-based noninvasive glucose monitoring: A proof-of-concept study. Anal. Chem..

[6] Bandodkar A.J., Jia W., Wang J. (2015). Tattoo-based wearable electrochemical devices: A review. Electroanalysis.

[7] Miller P.R., Narayan R.J., Polsky R. (2016). Microneedle-based sensors for medical diagnosis. J. Mater. Chem. B.

[8] Li H., Wu G., Weng Z., Sun H., Nistala R., Zhang Y. (2021). Microneedle-based potentiometric sensing system for continuous monitoring of multiple electrolytes in skin interstitial fluids. ACS Sens..

[9] Kim J., Jeerapan I., Imani S., Cho T.N., Bandodkar A., Cinti S., Mercier P.P., Wang J. (2016). Noninvasive alcohol monitoring using a wearable tattoo-based iontophoretic-biosensing system. ACS Sens..

[10] Wang M., Yang Y., Min J., Song Y., Tu J., Mukasa D., Ye C., Xu C., Heflin N., McCune J.S. (2022). A wearable electrochemical biosensor for the monitoring of metabolites and nutrients. Nat. Biomed. Eng..

[11] Bandodkar A.J., Hung V.W.S., Jia W., Valdés-Ramírez G., Windmiller J.R., Martinez A.G., Ramírez J., Chan G., Kerman K., Wang J. (2013). Tattoo-based potentiometric ion-selective sensors for epidermal ph monitoring. Analyst.

[12] Parrilla M., Cuartero M., Crespo G.A. (2019). Wearable potentiometric ion sensors. TrAC Trends Anal. Chem..

[13] Parrilla M., Detamornrat U., Domínguez-Robles J., Donnelly R.F., De Wael K. (2022). Wearable hollow microneedle sensing patches for the transdermal electrochemical monitoring of glucose. Talanta.

[14] Zhang B.L., Yang Y., Zhao Z.Q., Guo X.D. (2020). A gold nanoparticles deposited polymer microneedle enzymatic biosensor for glucose sensing. Electrochim. Acta.

[15] Cheng Y., Gong X., Yang J., Zheng G., Zheng Y., Li Y., Xu Y., Nie G., Xie X., Chen M. (2022). A touch-actuated glucose sensor fully integrated with microneedle array and reverse iontophoresis for diabetes monitoring. Biosens. Bioelectron..

[16] Bollella P., Sharma S., Cass A.E.G., Antiochia R. (2019). Microneedle-based biosensor for minimally-invasive lactate detection. Biosens. Bioelectron..

[17] Mohan A.M.V., Windmiller J.R., Mishra R.K., Wang J. (2017). Continuous minimally-invasive alcohol monitoring using microneedle sensor arrays. Biosens. Bioelectron..

[18] Senel M., Dervisevic M., Voelcker N.H. (2019). Gold microneedles fabricated by casting of gold ink used for urea sensing. Mater. Lett..

[19] Wu Y., Tehrani F., Teymourian H., Mack J., Shaver A., Reynoso M., Kavner J., Huang N., Furmidge A., Duvvuri A. (2022). Microneedle aptamer-based sensors for continuous, real-time therapeutic drug monitoring. Anal. Chem..

[20] Goud K.Y., Moonla C., Mishra R.K., Yu C., Narayan R., Litvan I., Wang J. (2019). Wearable electrochemical microneedle sensor for continuous monitoring of levodopa: Toward parkinson management. ACS Sens..

[21] Mishra R.K., Vinu Mohan A.M., Soto F., Chrostowski R., Wang J. (2017). A microneedle biosensor for minimally-invasive transdermal detection of nerve agents. Analyst.

[22] Teymourian H., Moonla C., Tehrani F., Vargas E., Aghavali R., Barfidokht A., Tangkuaram T., Mercier P.P., Dassau E., Wang J. (2020). Microneedle-based detection of ketone bodies along with glucose and lactate: Toward real-time continuous interstitial fluid monitoring of diabetic ketosis and ketoacidosis. Anal. Chem..

[23] Wang J., Wang H., Lai L., Li Y. (2021). Preparation of microneedle array mold based on mems lithography technology. Micromachines.

[24] Gill H.S., Prausnitz M.R. (2007). Coated microneedles for transdermal delivery. J. Control. Release.

[25] Omatsu T., Chujo K., Miyamoto K., Okida M., Nakamura K., Aoki N., Morita R. (2010). Metal microneedle fabrication using twisted light with spin. Opt. Express.

[26] Mansoor I., Liu Y., Häfeli U.O., Stoeber B. (2013). Arrays of hollow out-of-plane microneedles made by metal electrodeposition onto solvent cast conductive polymer structures. J. Micromech. Microeng..

[27] Kim J.D., Kim M., Yang H., Lee K., Jung H. (2013). Droplet-born air blowing: Novel dissolving microneedle fabrication. J. Control. Release.

[28] McGrath M.G., Vucen S., Vrdoljak A., Kelly A., O’Mahony C., Crean A.M., Moore A. (2014). Production of dissolvable microneedles using an atomised spray process: Effect of microneedle composition on skin penetration. Eur. J. Pharm. Biopharm..

[29] Martanto W., Moore J.S., Kashlan O., Kamath R., Wang P.M., O’Neal J.M., Prausnitz M.R. (2006). Microinfusion using hollow microneedles. Pharm. Res..

[30] Silvestre S.L., Araújo D., Marques A.C., Pires C., Matos M., Alves V., Martins R., Freitas F., Reis M.A.M., Fortunato E. (2020). Microneedle arrays of polyhydroxyalkanoate by laser-based micromolding technique. ACS Appl. Bio. Mater..

[31] Wang Q.L., Zhu D.D., Chen Y., Guo X.D. (2016). A fabrication method of microneedle molds with controlled microstructures. Mater. Sci. Eng. C.

[32] Krieger K.J., Bertollo N., Dangol M., Sheridan J.T., Lowery M.M., O’Cearbhaill E.D. (2019). Simple and customizable method for fabrication of high-aspect ratio microneedle molds using low-cost 3d printing. Microsyst. Nanoeng..

[33] Cass A.E.G., Sharma S., Thompson R.B., Fierke C.A. (2017). Chapter fifteen—Microneedle enzyme sensor arrays for continuous in vivo monitoring. Methods in Enzymology.

[34] Zhou Y., Danbolt N.C. (2014). Glutamate as a neurotransmitter in the healthy brain. J. Neural Transm..

[35] Iovino L., Tremblay M.E., Civiero L. (2020). Glutamate-induced excitotoxicity in parkinson’s disease: The role of glial cells. J. Pharm. Sci..

[36] Wang R., Reddy P.H. (2017). Role of glutamate and nmda receptors in alzheimer’s disease. J.Alzheimer’s Dis..

[37] Wu C., Barkova D., Komarova N., Offenhäusser A., Andrianova M., Hu Z., Kuznetsov A., Mayer D. (2022). Highly selective and sensitive detection of glutamate by an electrochemical aptasensor. Anal. Bioanal. Chem..

[38] Wang W., He Y., Gao Y., Gao H., Deng L., Gui Q., Cao Z., Yin Y., Feng Z. (2022). A peptide aptamer based electrochemical amperometric sensor for sensitive l-glutamate detection. Bioelectrochemistry.

[39] Wang Y., Mishra D., Bergman J., Keighron J.D., Skibicka K.P., Cans A.-S. (2019). Ultrafast glutamate biosensor recordings in brain slices reveal complex single exocytosis transients. ACS Chem. Neurosci..

[40] Özel R.E., Ispas C., Ganesana M., Leiter J.C., Andreescu S. (2014). Glutamate oxidase biosensor based on mixed ceria and titania nanoparticles for the detection of glutamate in hypoxic environments. Biosens. Bioelectron..

[41] Yang X.-K., Zhang F.-L., Wu W.-T., Tang Y., Yan J., Liu Y.-L., Amatore C., Huang W.-H. (2021). Quantitative nano-amperometric measurement of intravesicular glutamate content and its sub-quantal release by living neurons. Angew. Chem. Int. Ed..

[42] Bermingham K.P., Doran M.M., Bolger F.B., Lowry J.P. (2022). Design optimisation and characterisation of an amperometric glutamate oxidase-based composite biosensor for neurotransmitter l-glutamic acid. Anal. Chim. Acta.

[43] Windmiller J.R., Valdés-Ramírez G., Zhou N., Zhou M., Miller P.R., Jin C., Brozik S.M., Polsky R., Katz E., Narayan R. (2011). Bicomponent microneedle array biosensor for minimally-invasive glutamate monitoring. Electroanalysis.

[44] Benveniste H., Drejer J., Schousboe A., Diemer N.H. (1984). Elevation of the extracellular concentrations of glutamate and aspartate in rat hippocampus during transient cerebral ischemia monitored by intracerebral microdialysis. J. Neurochem..

[45] Bai W., Zhou Y.G. (2017). Homeostasis of the intraparenchymal-blood glutamate concentration gradient: Maintenance, imbalance, and regulation. Front. Mol. Neurosci..

[46] Kim G., Kim H., Kim I.J., Kim J.R., Lee J.I., Ree M. (2009). Bacterial adhesion, cell adhesion and biocompatibility of nafion films. J. Biomater. Sci. Polym. Ed..

[47] Turner R.F.B., Harrison D.J., Rojotte R.V. (1991). Preliminary in vivo biocompatibility studies on perfluorosulphonic acid polymer membranes for biosensor applications. Biomaterials.

[48] Drew S.M., Belzer T. (2023). An amperometric glucose biosensor composed of prussian blue, nafion, and glucose oxidase studied by flow injection analysis. J. Chem. Educ..

[49] Haghighi B., Tabrizi M.A. (2011). Direct electron transfer from glucose oxidase immobilized on a nano-porous glassy carbon electrode. Electrochim. Acta.

[50] Lee W., Jeong S.-H., Lim Y.-W., Lee H., Kang J., Lee H., Lee I., Han H.-S., Kobayashi S., Tanaka M. (2021). Conformable microneedle ph sensors via the integration of two different siloxane polymers for mapping peripheral artery disease. Sci. Adv..

[51] Zhao L., Wen Z., Jiang F., Zheng Z., Lu S. (2020). Silk/polyols/god microneedle based electrochemical biosensor for continuous glucose monitoring. RSC Adv..

[52] Prass S., St-Pierre J., Klingele M., Friedrich K.A., Zamel N. (2021). Hydrogen oxidation artifact during platinum oxide reduction in cyclic voltammetry analysis of low-loaded pemfc electrodes. Electrocatalysis.

[53] Wu J., Fan X., Liu J., Luo Q., Xu J., Chen X. (2018). Promoter engineering of cascade biocatalysis for α-ketoglutaric acid production by coexpressing l-glutamate oxidase and catalase. Appl. Microbiol. Biotechnol..

[54] Maity D., Kumar R.T.R. (2019). Highly sensitive amperometric detection of glutamate by glutamic oxidase immobilized pt nanoparticle decorated multiwalled carbon nanotubes(mwcnts)/polypyrrole composite. Biosens. Bioelectron..

[55] Batra B., Kumari S., Pundir C.S. (2014). Construction of glutamate biosensor based on covalent immobilization of glutmate oxidase on polypyrrole nanoparticles/polyaniline modified gold electrode. Enzym. Microb. Technol..

[56] Hughes G., Pemberton R.M., Fielden P.R., Hart J.P. (2015). Development of a novel reagentless, screen-printed amperometric biosensor based on glutamate dehydrogenase and nad^+^, integrated with multi-walled carbon nanotubes for the determination of glutamate in food and clinical applications. Sens. Actuators B Chem..

[57] Soldatkina O.V., Soldatkin O.O., Kasap B.O., Kucherenko D.Y., Kucherenko I.S., Kurc B.A., Dzyadevych S.V. (2017). A novel amperometric glutamate biosensor based on glutamate oxidase adsorbed on silicalite. Nanoscale Res Lett..

[58] Yang L., Bai R., Xie B., Zhuang N., Lv Z., Chen M., Dong W., Zhou J., Jiang M. (2023). A biosensor based on oriented immobilization of an engineered l-glutamate oxidase on a screen-printed microchip for detection of l-glutamate in fermentation processes. Food Chem..

[59] Zeynaloo E., Yang Y.-P., Dikici E., Landgraf R., Bachas L.G., Daunert S. (2021). Design of a mediator-free, non-enzymatic electrochemical biosensor for glutamate detection. Nanomed. NBM.

[60] Batra B., Pundir C.S. (2013). An amperometric glutamate biosensor based on immobilization of glutamate oxidase onto carboxylated multiwalled carbon nanotubes/gold nanoparticles/chitosan composite film modified au electrode. Biosens. Bioelectron..

[61] Liang B., Zhang S., Lang Q., Song J., Han L., Liu A. (2015). Amperometric l-glutamate biosensor based on bacterial cell-surface displayed glutamate dehydrogenase. Anal. Chim. Acta.

[62] Martínez-Periñán E., Domínguez-Saldaña A., Villa-Manso A.M., Gutiérrez-Sánchez C., Revenga-Parra M., Mateo-Martí E., Pariente F., Lorenzo E. (2023). Azure a embedded in carbon dots as nadh electrocatalyst: Development of a glutamate electrochemical biosensor. Sens. Actuators B Chem..

[63] Liu J., Fan Y., Chen G., Liu Y. (2021). Highly sensitive glutamate biosensor based on platinum nanoparticles decorated mxene-ti3c2tx for l-glutamate determination in foodstuffs. LWT.

[64] Wang X., Duan J., Cai Y., Liu D., Li X., Dong Y., Hu F. (2020). A modified nanocomposite biosensor for quantitative l-glutamate detection in beef. Meat Sci..

[65] Mentana A., Nardiello D., Palermo C., Centonze D. (2020). Accurate glutamate monitoring in foodstuffs by a sensitive and interference-free glutamate oxidase based disposable amperometric biosensor. Anal. Chim. Acta.

