# Supplementary material for: A Facile Method for the Fabrication of the Microneedle Electrode and Its Application in the Enzymatic Determination of Glutamate
Source: Biosensors (Basel). 2023 Aug 18;13(8):828. doi: 10.3390/bios13080828 (PMC10452303; doi:10.3390/bios13080828)
Supplement: Supplementary file 1 [file biosensors-13-00828-s001.zip › biosensors-2491483-supplementary.pdf]

Supplementary material

A Facile Method for the Fabrication of the Microneedle Electrode and its Application in the Enzymatic Determination of Glutamate

Mahmoud Amouzadeh Tabrizi

Electronic Technology Department, Universidad Carlos III de Madrid, 28911Leganés, Spain; mamouzad@ing.uc3m.es or mahmoud.tabrizi@gmail.com

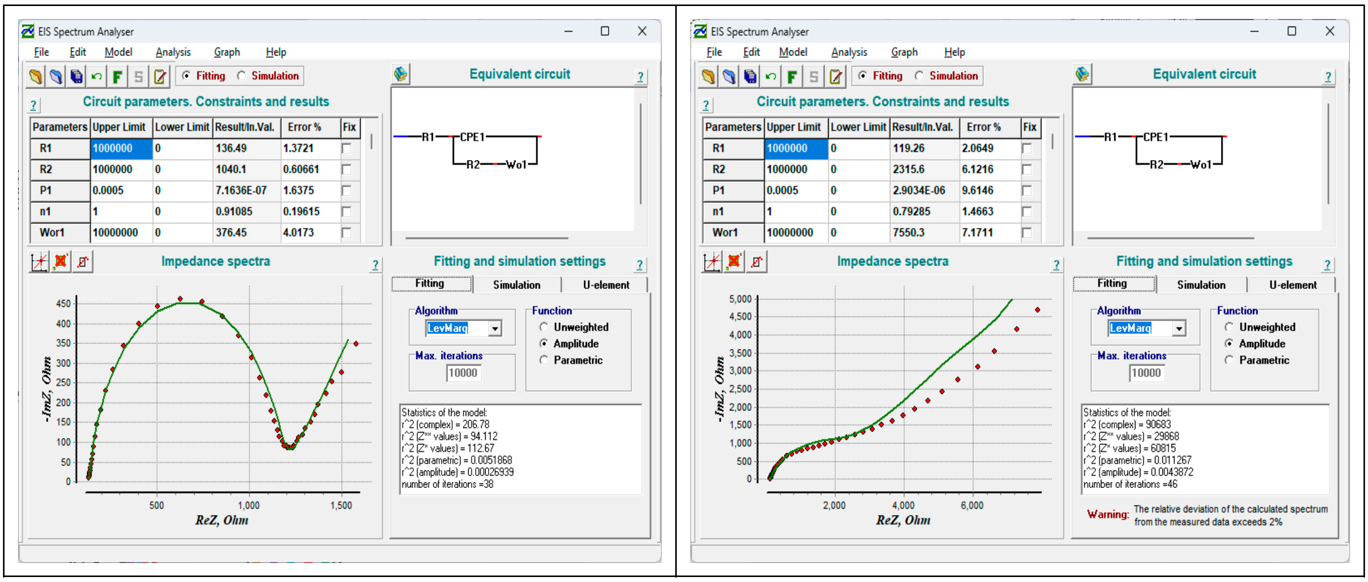

Figure S1. Images of the modeling results with values of the different parameters
